# Tumor Suppressor Role of Wild-Type P53-Dependent Secretome and Its Proteomic Identification in PDAC

**DOI:** 10.3390/biom12020305

**Published:** 2022-02-13

**Authors:** Giovanna Butera, Marcello Manfredi, Alessandra Fiore, Jessica Brandi, Raffaella Pacchiana, Veronica De Giorgis, Elettra Barberis, Virginia Vanella, Marilisa Galasso, Maria Teresa Scupoli, Emilio Marengo, Daniela Cecconi, Massimo Donadelli

**Affiliations:** 1 Department of Neurosciences, Biomedicine and Movement Sciences, Section of Biochemistry, University of Verona, 37134 Verona, Italy; giovanna.butera@univr.it (G.B.); alessandra.fiore@univr.it (A.F.); raffaella.pacchiana@univr.it (R.P.); marilisa.galasso@univr.it (M.G.); mariateresa.scupoli@univr.it (M.T.S.); 2 Department of Translational Medicine, University of Piemonte Orientale, 28100 Novara, Italy; marcello.manfredi@uniupo.it (M.M.); 20019924@studenti.uniupo.it (V.D.G.); elettra.barberis@uniupo.it (E.B.); virginia.vanella@uniupo.it (V.V.); 3 Center for Translational Research on Autoimmune and Allergic Diseases, University of Piemonte Orientale, 28100 Novara, Italy; emilio.marengo@uniupo.it; 4 ISALIT, Spin-off at the University of Piemonte Orientale, 28100 Novara, Italy; 5 Department of Biotechnology, University of Verona, 37134 Verona, Italy; jessica.brandi@univr.it (J.B.); daniela.cecconi@univr.it (D.C.); 6 Department of Medicine, Section of Hematology, University of Verona, 37134 Verona, Italy; 7 Research Center LURM, Interdepartmental Laboratory of Medical Research, University of Verona, 37134 Verona, Italy; 8 Department of Sciences and Technological Innovation, University of Piemonte Orientale, 28100 Novara, Italy

**Keywords:** wild-type p53, secretome, pancreatic ductal adenocarcinoma, onco-suppressor gene

## Abstract

The study of the cancer secretome is gaining even more importance in cancers such as pancreatic ductal adenocarcinoma (PDAC), whose lack of recognizable symptoms and early detection assays make this type of cancer highly lethal. The wild-type p53 protein, frequently mutated in PDAC, prevents tumorigenesis by regulating a plethora of signaling pathways. The importance of the p53 tumor suppressive activity is not only primarily involved within cells to limit tumor cell proliferation but also in the extracellular space. Thus, loss of p53 has a profound impact on the secretome composition of cancer cells and marks the transition to invasiveness. Here, we demonstrate the tumor suppressive role of wild-type p53 on cancer cell secretome, showing the anti-proliferative, apoptotic and chemosensitivity effects of wild-type p53 driven conditioned medium. By using high-resolution SWATH-MS technology, we characterized the secretomes of p53-deficient and p53-expressing PDAC cells. We found a great number of secreted proteins that have known roles in cancer-related processes, 30 of which showed enhanced and 17 reduced secretion in response to p53 silencing. These results are important to advance our understanding on the link between wt-p53 and cancer microenvironment. In conclusion, this approach may detect a secreted signature specifically driven by wild-type p53 in PDAC.

## 1. Introduction

Pancreatic ductal adenocarcinoma (PDAC) is one of the most invasive solid tumors and its incidence is increasing worldwide [1,2]. The molecular complexity and the absence of early specific symptoms, as well as the efficient methods for its early detection, ensure that only about 20% of pancreatic cancers are detected early enough to be surgically resectable [3]. In fact, around 50% of diagnosed PDAC patients present with metastatic disease [3]. One of the most important proteins involved in inducing cell cycle arrest, DNA repair or programmed cell death is the tumor suppressor protein p53, which controls a wide range of cellular biological processes to prevent the outgrowth of aberrant cells [4]. In response to various cellular insults that include oxidative stress and oncogenic signaling, p53 is activated and acts as a sequence-specific transcription factor [5,6]. P53′s potency in suppressing abnormal clonal outgrowth is inhibited in many cancer types including PDAC, in which p53 dysfunctions are frequent [7,8,9]. The most common p53 mutations are missense mutations in the DNA-binding domain (DBD) [10]. In addition to the loss of its function, mutant p53 proteins may exert a dominant negative effect, inhibiting the function of wild-type p53 (WTp53) by preventing it from binding to the promoter of its target genes, and may have gain-of-function activities, exhibiting oncogenic properties [11,12]. Several studies highlight the contrasting roles between WT and mutant p53. Our recent studies reveal that in contrast to the WTp53 roles, mutant p53 induces aberrant alterations in cancer metabolism and reactive oxygen species (ROS) production, contributing to pancreas cancer development and chemoresistance [13,14,15].

A growing importance is emerging to consider not only the intracellular roles of p53 but also its extracellular impact in the regulation of the cancer microenvironment, which is important also for the identification of targeted cancer therapies and novel serum biomarkers. Our previous study reveals the functional effect of hot-spot p53 mutants on cancer cell secretome which promote oncogenic roles as chemoresistance, cell migration and epithelial-mesenchymal transition [16,17].

In the present study, we investigate the role of WTp53-driven secretome of PDAC cells, demonstrating the functional tumor suppressive effect of WTp53 on cancer cell secretome. Furthermore, we characterize the secretomes of p53-deficient and p53-expressing Paca3 human PDAC cells by using high-resolution Sequential Window Acquisition of all Theoretical Mass Spectra (SWATH-MS) technology. We identify 30 hypersecreted proteins and 17 proteins with reduced secretion in response to p53 silencing. These results highlight the tumor suppressor effect of WTp53 on the pancreatic cancer microenvironment and suggest a WTp53-dependent PDAC secretome, providing the basis for the identification of secreted protein biomarkers specifically driven by WTp53 in PDAC.

## 2. Materials and Methods

### 2.1. Drug

Gemcitabine (2′,2′-difluoro-2′-deoxycytidine; GEM) was provided by Accord Healthcare (Milan, Italy) and solubilized in sterile bidistilled water.

### 2.2. Cell Cultures

Pancreatic adenocarcinoma AsPC-1 (p53-null), PaCa3 (WTp53), Hs 776T (WTp53), and normal human pancreatic duct epithelial (HPDE1) (WTp53) cells were grown in RPMI 1640 (Life Technologies, Milan, Italy). Culture media were supplemented with 10% FBS, and 50 μg/mL gentamicin sulfate (BioWhittaker, Lonza, Bergamo, Italy). PaCa3, Hs 776T and HPDE1 cells were kindly provided by Dr. Aldo Scarpa (University of Verona, Italy) and AsPC-1 was purchased by ATCC (Manassas, VA, USA). The adherent cells were incubated at 37 °C with 5% CO_2_.

### 2.3. Transient Transfection Assay

AsPC-1 and PaCa3 cells were seeded in 96-well or in 6-well plates. Wild-type ectopic expression in AsPC-1 p53-null cancer cells was obtained by transfecting pCMV-wild-type p53 expression vectors or its relative negative control. WTp53 protein expression in PaCa3 cells was transiently knocked-down by transfection with commercial siRNA smart pool of three oligonucleotides (sip53) transiently targeting p53 (Santa Cruz Biotech, Dallas, TX, USA; sc-29435). Transfections were carried out using Lipofectamine 3000 (Thermo Fisher Scientific, Milan, Italy) for 48 h, according to the manufacturer’s instructions.

### 2.4. Cell Proliferation Assay

PDAC cells were seeded on 96-well plates (9 × 10^3^ cells/well). Forty-eight hours later, cell growth was measured by Crystal Violet assay (Sigma, Milan, Italy) according to the manufacturer’s protocol, and the absorbance was measured by spectrophotometric analysis (A595 nm).

### 2.5. Apoptosis Assay

The cells were seeded on 96-well plates. Forty-eight hours later, cells were fixed with 2% paraformaldehyde in PBS for 10 min at room temperature, washed with PBS, and then stained with annexinV/FITC (Bender MedSystem, Milan, Italy) in binding buffer (10 mM HEPES/NaOH pH 7.4, 140 mM NaCl, and 2.5 mM CaCl_2_) for 10 min in the dark. The fluorescence was measured using a multimode plate reader (Ex485 nm and Em535 nm) (GENios Pro, Tecan, Milan, Italy). The values were normalized on cell proliferation by Crystal Violet assay.

### 2.6. Autophagosome Formation Assay

The cells were stained with the fluorescent probe monodansylcadaverine (MDC; Sigma, Milan, Italy) to quantify the induction of autophagy. Briefly, cells were seeded on 96-well plates and, 48 h later, cells were incubated in culture medium containing 50 μM MDC at 37 °C for 15 min. Cells were then washed with Hanks buffer (20 mM Hepes pH 7.2, 10 mM glucose, 118 mM NaCl, 4.6 mM KCl, and 1 mM CaCl_2_) and fluorescence was measured using a multimode plate reader (Ex340 nm and Em535 nm) (GENios Pro, Tecan, Milan, Italy). The values were normalized on cell proliferation by Crystal Violet assay.

### 2.7. Wound-Closure Cell Migration Assay

AsPC-1 cells were seeded in six-well plate (9 × 105 cells/well). A scratch was made across the center of the AsPC1 p53-null monolayer cells. The cells were incubated with conditioned medium (CM) released by transfected AsPC-1 cells for 48 h. Cell migration was observed in time-lapse (EVOS). The images were captured every 2 h for 48 h and were further analyzed quantitatively using NIH ImageJ computing software (http://rsb.info.nih.gov/nih-image/ accessed on 10 February 2022 ). Migration ability as relative migration distance (RMD) was evaluated using the following formula: RMD (%) = 100 (A − B)/A, with A and B representing the width of cell scratches at time 0 and after 48 h of incubation, respectively.

### 2.8. Immunoblot Analysis

The cells were harvested, washed in PBS, and solubilized in lysis buffer in the presence of phosphatase and protease inhibitors (50 mM Tris–HCl pH 8, 150 mM NaCl, 1% Igepal CA-630, 0.5% Na-Doc, 0.1% SDS, 1 mM Na_3_VO_4_, 1 mM NaF, 2.5 mM EDTA, 1 mM PMSF, and 1× protease inhibitor cocktail). After incubation on ice for 30 min, the lysates were centrifuged at 14,000× *g* for 10 min at 4 °C and the supernatant fractions were used for Western blot analysis. The protein extracts (30 μg/lane) were resolved on a 12% SDS-polyacrylamide gel and electro-blotted onto PVDF membranes (Millipore, Milan, Italy). The membranes were blocked in 5% low-fat milk in TBST (50 mM Tris pH 7.5, 0.9% NaCl, 0.1% Tween 20) for 1 h at room temperature and probed overnight at 4 °C with a mouse polyclonal anti-p53 (1:2000) (Santa Cruz, #sc-263), rabbit monoclonal anti-glyceraldehyde 3-phosphate dehydrogenase (GAPDH) (1:1000) (Cell Signaling, #5174S), rabbit monoclonal anti-β-Actin (1:1000, 13E5, #5125, Cell Signaling) antibodies. Horseradish peroxidase conjugated anti-mouse or anti-rabbit IgGs (1:8000 in blocking solution) (Upstate Biotechnology, Milan, Italy) were used as secondary antibodies. Immunodetection was carried out using chemiluminescent substrates (Amersham Pharmacia Biotech, Milan, Italy) and recorded using a HyperfilmECL (Amersham Pharmacia Biotech, Milan, Italy). The ECL (Enhanced ChemiLuminescence) results were scanned and the amount of each protein band was quantified using NIH Image J software (Version 1.53f51, National Institutes of Health (NIH), Bethesda, MA, USA).

### 2.9. Protein Extraction from Conditioned Medium (CM)

The day after transient transfection, PaCa3 cells were washed six times in PBS and then incubated in serum-free RPMI for 22 h. Cell viability, as determined with 0.4% trypan blue solution (Thermo Fischer Scientific, Milan, Italy ), was higher than 95%. The media containing secreted proteins were collected by centrifugation at 1000× *g* for 10 min to pellet floating cells and were defined as conditioned media (CM). After the addition of 1× protease inhibitor cocktail (Roche, Basel, Switzerland ), CM were centrifuged again at 17,000× *g* for 20 min at 4 °C to pellet the remaining cell debris. The proteins in the CM were precipitated overnight at −20 °C with 4 volumes of ice-cold acetone. The pellets were then collected by centrifugation at 17,000× *g* for 20 min at 4 °C and then resuspended in 100 mM ammonium bicarbonate (NH_4_HCO_3_). The protein concentration was determined using BCA protein assay (Sigma, Milan, Italy ).

### 2.10. Proteomic Analysis

Comparative secretome analysis between wild-type and knock-down p53 PaCa3 cells was performed by analyzing three biological replicates, as previously reported [16]. Briefly, proteins were reduced with dithiothreitol (200 mM DTT stock solution) (Sigma) at 90 °C for 20 min, alkylated with 10 µL of Cysteine Blocking Reagent (Iodoacetamide, IAM, 200 mM; Sigma) for 1 h at room temperature in the dark and digested with trypsin (Promega, Sequence Grade) overnight at 37 °C. Trypsin activity was stopped by adding 2 µL of neat formic acid and the digests were dried by Speed Vacuum [18]. The digested proteins were analyzed on a micro-LC Eksigent Technologies (Dublin, OH, USA) interfaced to a 5600+ TripleTOF mass spectrometer system (AB Sciex, Concord, ON, Canada) as previously described. For identification purposes, the samples were subjected to data dependent analysis (DDA) while the quantification was performed using a cyclic data independent analysis (DIA) [19]. The MS data were acquired with Analyst TF 1.7 (AB SCIEX, Concord, ON, Canada). Two DDA and three DIA acquisitions were performed. The DDA files were searched using Protein Pilot software v. 4.2 (AB SCIEX, Concord, ON, Canada) and Mascot v. 2.4 (Matrix Science Inc., Boston, MA, USA). Trypsin as digestion enzyme was specified for both the software. For Mascot we used two missed cleavages, the instrument was set to ESI-QUAD-TOF, and the following modifications were specified for the search: carbamidomethyl of cysteines as fixed modification and oxidized methionine as variable modification. A search tolerance of 0.08 Da was specified for the peptide mass tolerance, and 10 ppm for the MS/MS tolerance. The charges of the peptides to search for were set to 2 +, 3 +, and 4 +, and the search was set on monoisotopic mass. The UniProt Swiss-Prot reviewed database containing human proteins (version 2015.07.07, containing 42131 sequence entries) was used and a target-decoy database search was performed. False Discovery Rate was fixed at 1% [20]. The label-free quantification was performed using Skyline 3.1, an open-source software project (http://proteome.gs.washington.edu/software/skyline accessed on 10 February 2022 ) [21]. For statistical analysis of quantitative differences of proteins and peptides between samples, MSstats (v.2.0), an open-source R-based package [22] was used.

### 2.11. Bioinformatics Evaluation of Proteomics Data

Bioinformatic analysis was used to extract biological information. The hyper- and hyposecreted proteins were analyzed using the STRING database (v.11.0) (http://string-db.org accessed on 10 February 2022), to predict protein-protein interactions [23]. Whilst pathways enrichment and upstream regulators analyses were performed by using the Ingenuity Pathways Analysis (IPA) software (Qiagen, Redwood City, CA, USA).

### 2.12. Statistical Analysis

ANOVA analysis was performed by GraphPad Prism 5 software (GraphPad Software, San Diego, CA, USA). *p* value < 0.05 was indicated as being statistically significant. Values are the means of three independent experiments (±SD).

## 3. Results

### 3.1. Cancer Cell Secretome of Wild-Type P53 PDAC Cells Exhibits Suppressor Roles

**Figure 1:**
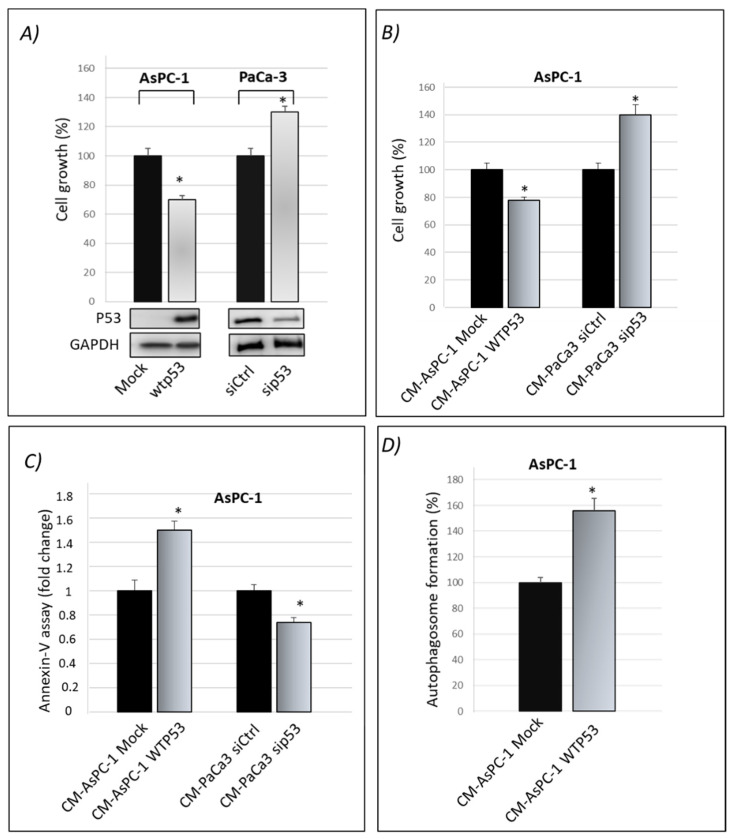
Cancer cell secretome driven by wild-type p53 exhibits oncosuppressor roles. (**A**) Cell growth was measured by Cristal Violet assay in p53-null AsPC-1 cells transfected for overexpression of wtp53 and in PaCa3 after knocking-down of endogenous p53 to verify the transfection efficiency. Accompanying Western blotting of p53 and of GAPDH for control loading are reported. Statistical analysis * *p* < 0.05 wtp53 vs. Mock AsPC-1; sip53 vs. siCtrl PaCa3. (**B**) Cell growth was measured by Cristal Violet assay in untransfected p53-null AsPC-1 cells cultivated with WTp53-CM from AsPC1 or with sip53-CM from PaCa3, each one compared by their respective control. Statistical analysis * *p* < 0.05 CM-AsPC-1 WTp53 vs. CM-Mock; CM-PaCa3 sip53 vs. CM-Paca3 siCtrl. (**C**) Apoptosis was determined by the annexinV/FITC binding assay in AsPC-1 cultivated with wtp53-derived CM of AsPC-1 or with sip53-CM from PaCa3, each one compared by their respective control. Statistical analysis * *p* < 0.05 CM-AsPC-1 WTp53 vs. CM-Mock; CM-PaCa3 sip53 vs. CM-Paca3 siCtrl. (**D**) Autophagosome formation assay was determined by intracellular staining using the MDC probe in AsPC-1 cultivated with WTp53-derived CM of AsPC-1. Statistical analysis * *p* < 0.05 CM-AsPC-1wtp53 vs. CM-Mock.

We previously published that gain-of-function mutant p53 isoforms exert their hyper-proliferative effects on cancer cells also through the alteration of their secretome [16]. Here, we aimed to study whether WTp53 may exhibit suppressor roles through its influence on the secretome of PDAC cells, in accordance with the tumor suppressor role of WTp53. By using the previously published approach [16], we induced the exogenous expression of WTp53 in p53-null AsPC-1 PDAC cells by using liposome-mediated transient transfection assay. In WTp53 PaCa3 cells, the endogenous p53 protein expression was transiently knocked down by transfection with siRNA. Forty-eight hours later, we checked the effective overexpression of p53 in AsPC-1 or knocking-down of WTp53 in PaCa3 cells by Western blotting and functionally analyzed their effect on cell growth induced by the presence or lack of wild-type p53, compared with their respective control (Figure 1A). Subsequently, AsPC-1 or PaCa3 transfected cells were washed in PBS and then incubated in fresh culture medium for further 22 h to accumulate secreted proteins. The conditioned medium (CM) released by transfected AsPC-1 or PaCa3 cells was transferred to untransfected p53-null AsPC-1 cells, which were thus cultivated for 48 h with the secretome driven by the presence or absence of wild-type p53. This allowed us to study the functional effects of the secretome driven by wild-type p53 overexpression or by p53 knock-down, each one compared by their respective control. In accordance with the tumor suppressor role of wild-type p53, Figure 1B,C show that p53-driven secretome is able to inhibit cell growth and promote apoptosis in AsPC-1 cells, as compared to its negative mock control. On the contrary, the conditioned medium of PaCa3 with silenced p53, is able to promote hyper-proliferative effects and to inhibit cell death of AsPC-1 cells, as compared to its negative control. The absence of extracellular p53 in WTp53-driven CM of AsPC-1 was previously proved by Western blotting [16] and then further confirmed by mass spectrometry analysis in both AsPC-1 and PaCa3 cells. Furthermore, since p53 is able to transactivate autophagy-related genes and to induce autophagy flux [24,25], we wondered whether even the conditioned medium driven by p53 can sustain autophagy. Figure 1D shows that WTp53-driven secretome promotes autophagic vesicles. Specifically, the conditioned medium released by transfected AsPC-1 expressing WTp53 is able to promote autophagosome formation in untransfected AsPC-1 cells. Altogether, these data provide evidence that WTp53 influences the secretion of proteins or other molecules that can functionally contribute to the regulation of cell growth and cell death-related phenomena, such as apoptosis and autophagy responses.

### 3.2. Wild-Type P53-Driven Secretome Counteracts Chemoresistance in PDAC Cells

**Figure 2:**
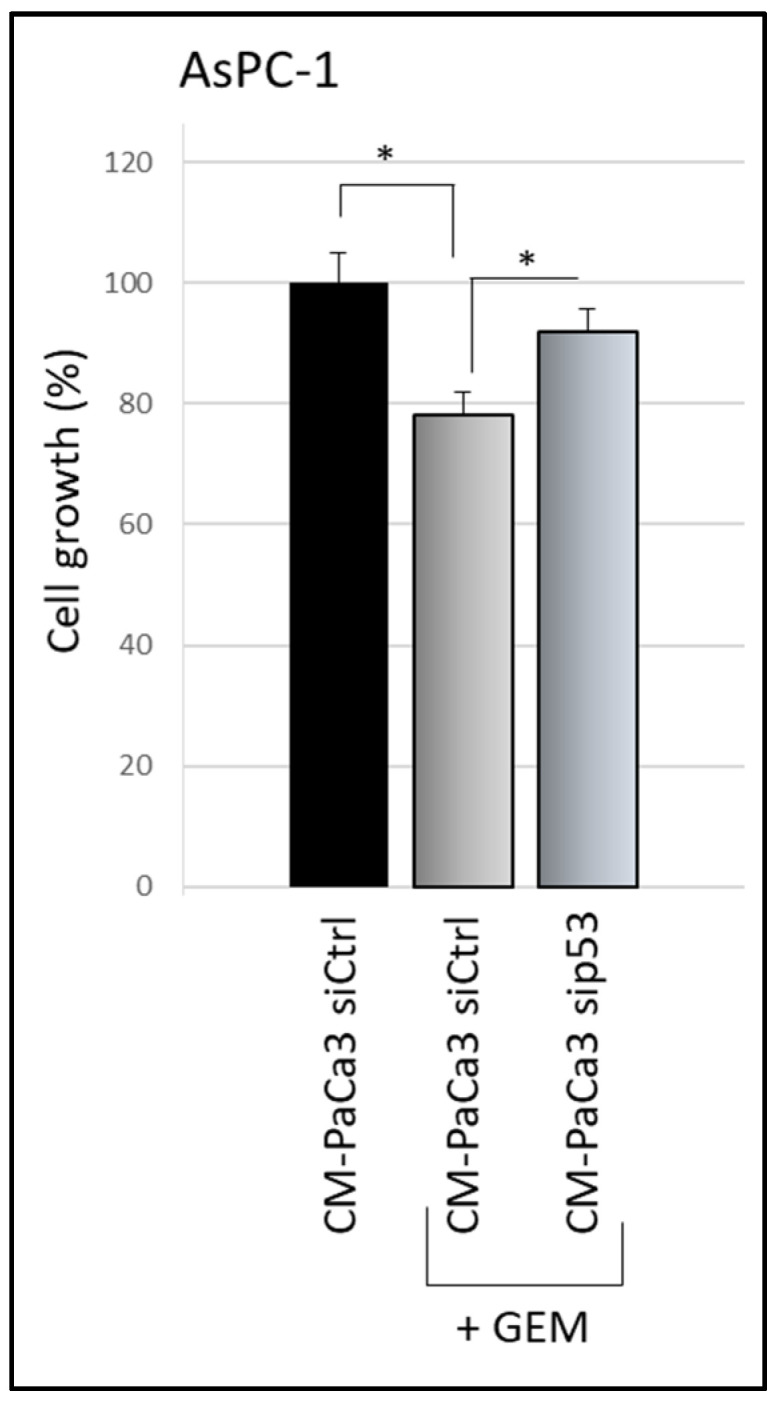
p53-driven secretome counteracts chemoresistance effects. Cell growth was measured by Cristal Violet assay in p53-null AsPC-1 cells cultivated with CM of sip53-PaCa3, or its control, and treated with 1 μM GEM for 48 h. Statistical analysis * *p* < 0.05 CM-Paca3 siCtrl vs. CM-Paca3 siCtrl + GEM; CM-PaCa3 sip53 + GEM vs. CM-Paca3 siCtrl + GEM.

A crucial oncosuppressor role induced by WTp53 is the stimulation of pancreatic cancer cell sensitivity to the treatment with the drug gemcitabine (GEM) [15,26]. Thus, we investigated whether cancer cell secretome driven by WTp53 is able to promote PDAC cell chemosensitivity. We observed that GEM treatment inhibited cell growth of AsPC-1 cells cultivated with CM derived by siCtrl-PaCa3 (Figure 2). Remarkably, GEM sensitivity of AsPC-1 cells was counteracted when cultivated in CM derived by sip53-PaCa3 cells (Figure 2), indicating a role for p53-driven secretome in the response to GEM. These data functionally demonstrate that wild-type p53 can exert its oncosuppressor role in PDAC cells through the regulation of its secretome, in line with the tumor suppressor role of p53 [27,28].

### 3.3. Mutp53-Driven Secretome Stimulates Cancer Cell Migration

**Figure 3:**
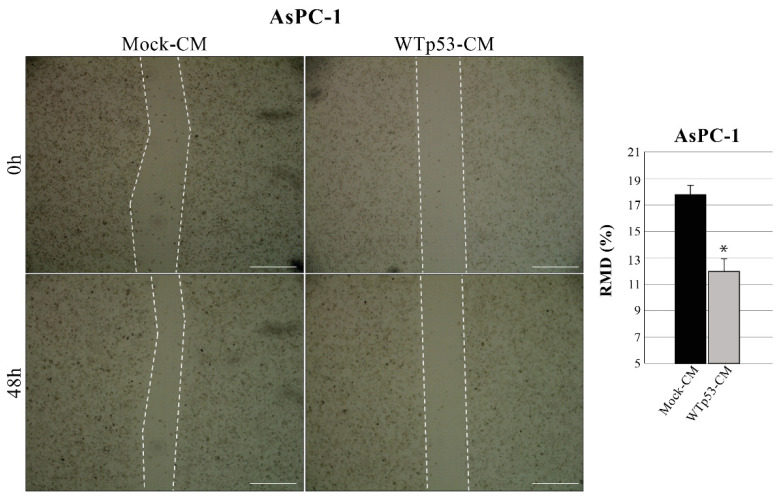
WTp53-driven secretome inhibits cancer cell migration. Wound closure cell assay on the confluent p53-null AsPC-1 cell monolayer cultivated with WTp53-derived conditioned medium (CM) as compared to CM derived from its mock control. A scratch was performed in the cell monolayer at time zero, after that we monitored cell migration for 48 h. The images were analyzed quantitatively by using ImageJ computing software. Migration ability expressed as relative migration distance (RMD) decreased in cells cultured with WTp53-derived CM. Statistical analysis *p < 0.05 CM-WTp53 vs. CM-Mock. Scale bar: 500 µm.

Since WTp53 has been reported to inhibit cancer cell migration, we also investigated whether WTp53-induced modulation of secretome can have a role in this phenomenon. Using the same methodological approach described in Figure 1, we discovered that WTp53-CM is able to reduce the migration rate of AsPC-1 cells, as compared to mock-CM control (mock-CM) (Figure 3). In particular, we observed a slower wound closure in p53-null AsPC-1 cells cultivated with WTp53-driven secretome, as compared to CM derived from its mock control. This result is in line with our previous data demonstrating that mutant p53-driven secretome stimulates PDAC cell migration and further confirms the tumor suppressor role of WTp53 and of its secretome in cancer cells.

### 3.4. Identification of Secreted Proteins from Wild-Type P53-Driven Secretome

After the investigation of the functional involvement of WTp53-driven secretome in the inhibition of PDAC cell growth and chemosensitivity to GEM, we aimed to identify the secreted proteins by WTp53 in PDAC cells. Thus, we compared the protein composition of the CM released by PaCa3 cells expressing WTp53 as compared to that of PaCa3 cells after p53 knock-down. To avoid protein cross-contamination by serum, cells were washed to remove DNA–liposome complexes and cultured for further 22 h to accumulate secreted proteins in serum-free culture medium. This serum-free culture period was previously identified as the maximum time period without delay of cell growth or signals of cell death, thus avoiding indiscriminate cellular lysis [16]. A peptide liquid chromatography separation followed by mass spectrometry analysis and database search with Protein Pilot and Mascot was then performed. SWATH-MS analyses were performed in triplicates for each analyzed sample, and they were imported in the Skyline software to perform the label-free quantification and the identification of hyper- and hyposecreted proteins. We identified and quantified 210 secreted proteins in both p53 knock-down and WT PaCa3 cells. Among them, the quantitative proteomic analysis reported the modulation (fold change > 1.3 and *p*-value < 0.05) of 47 secreted proteins, of which 30 were hyper secreted in p53 knock-down PaCa3 cells and 17 were hyposecreted (Table 1) compared to WTp53. Among the most hypersecreted proteins after p53-knock-down in PaCa3 cells there are GTP-binding protein Di-Ras2 (FC = 36.4), BCL-6 corepressor-like protein 1 (FC = 24.3), centrosomal protein of 78 kDa (FC = 23.7), glial fibrillary acidic protein (FC = 5.5), importin-5 (FC = 4.7), transferrin receptor protein 1 (FC = 3.8), isocitrate dehydrogenase (FC = 3.0), ATP-citrate synthase (FC = 2.9) and cathepsin B (FC = 2.6). On the other hand, p53 silencing caused a particular decrease in farnesyl pyrophosphate synthase (FC = 0.3), protein S100-A4 (FC = 0.3), transgelin-2 (FC = 0.3), tubulin beta-3 chain (FC = 0.3) and putative heat shock protein HSP 90-alpha A4 (FC = 0.3) in PaCa3 secretome.

Interestingly, these secreted protein from p53 knock-down and WTp53 PaCa3 cells were further compared with the secreted proteins of AsPC-1 cells GOF R175H and R273H mutp53 isoforms and their respective controls that we previously identified using the same method [16]. We identified several secreted proteins in common between CM-PaCa3 wild-p53 and CM-AsPC-1 GOF R175H and R273H. Specifically, the secreted heat shock protein beta-1 inhibited by WTp53 was hypersecreted by R175H-mutp53 AsPC-1 [16]; Cathepsin B and Transforming growth factor-beta-induced protein ig-h3 hyposecreted by WTp53 were hypersecreted in AsPc-1 cells GOF R273H; the heat shock protein HSP90-beta hypersecreted in WTp53was hyposecreted in AsPC-1 cells GOF R273H [16].

**Figure 4:**
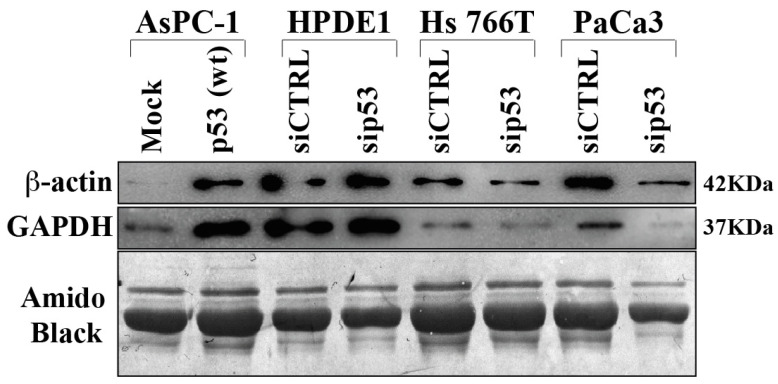
Immunoblot validation of p53-dependent hyposecreted proteins. p53-null AsPC-1 cells were transiently transfected with WTp53 or mock control plasmids, Hs 776T and PaCa3 WTp53-PDAC cell lines and HPDE1 human pancreas non-tumor cells were transiently transfected with siP53 to downregulate TP53; a scramble siRNA (siCTRL) was used as a control. A total of 48 h after transfection, cells were washed and further incubated for 22 h in serum-deprived media. Secreted proteins were precipitated overnight and p53-dependent GAPDH and ß-Actin protein expression was confirmed by immunoblot. Amido black staining was used as loading control.

In order to validate some differentially secreted proteins we performed Western bot analysis extending the evaluation also to other PDAC cell lines and to normal human pancreatic duct epithelial (HPDE1) as control (Figure 4). The results obtained confirm that p53 is able to determine hypersecretion of GAPDH and beta-actin proteins, as revealed in p53-null AsPC-1 cells in which p53 was overexpressed. On the other side, p53 knock-down reduced GAPDH and beta-actin secretion in Hs 766T and PaCa3 PDAC cell lines. It is noteworthy that p53 silencing in HPDE1 did not determine any significant change in protein secretion, suggesting that the deep modulation of protein secretome in PDAC cells by p53 may be a cancer-associated phenomenon.

### 3.5. Dysregulated Pathways, Protein Interaction Networks and Upstream Regulators Related to Wtp53-Driven Secreted Proteins

**Figure 5:**
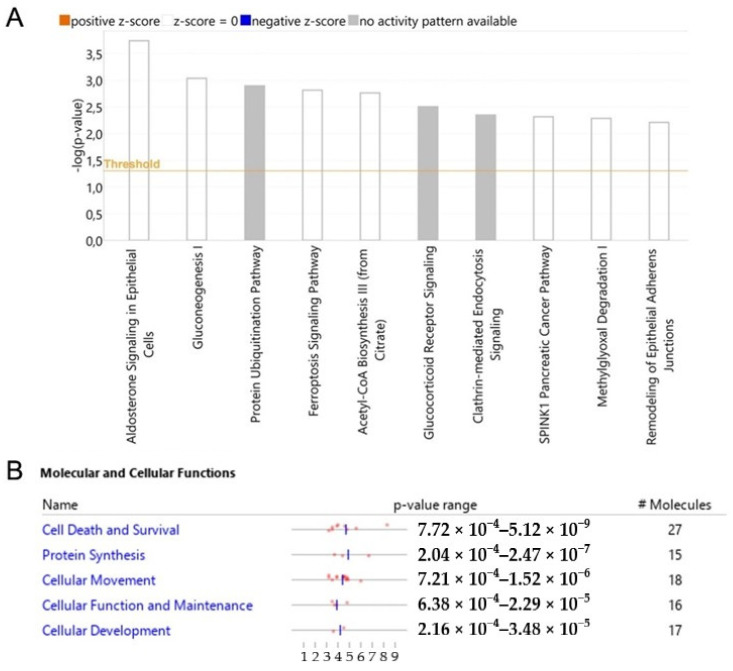
p53-driven canonical pathways and molecular and cellular functions. Bar-plot of canonical pathway significance (−log(*p*-value)) of altered secreted proteins in response to the silencing of p53 in PDAC (**A**). Activation/inhibition (z-score) was not predicted. Molecular and cellular functions with significance (*p*-value) and number of associated proteins (**B**).

We analyzed modulated proteins by bioinformatics tools to obtain a global overview of the interaction network by which WTp53 can control tumor development through secreted proteins in PDAC. Ingenuity Pathway Analysis (IPA) was employed to identify the main pathways and biological processes associated with the oncosuppressor role of p53. The canonical pathways linked to the silencing of p53 are mainly related to aldosterone signaling, gluconeogenesis, protein ubiquitination, ferroptosis signaling, acetyl-CoA biosynthesis, glucocorticoid receptor signaling, clathrin-mediated endocytosis, SPINK1 pancreatic cancer pathway, methylglyoxal degradation and remodeling of epithelial adherens junctions (Figure 5A). The analysis of the molecular and cellular functions revealed an involvement of proteins associated with cell death and survival, protein synthesis and cellular movement functions (Figure 5B).

**Figure 6:**
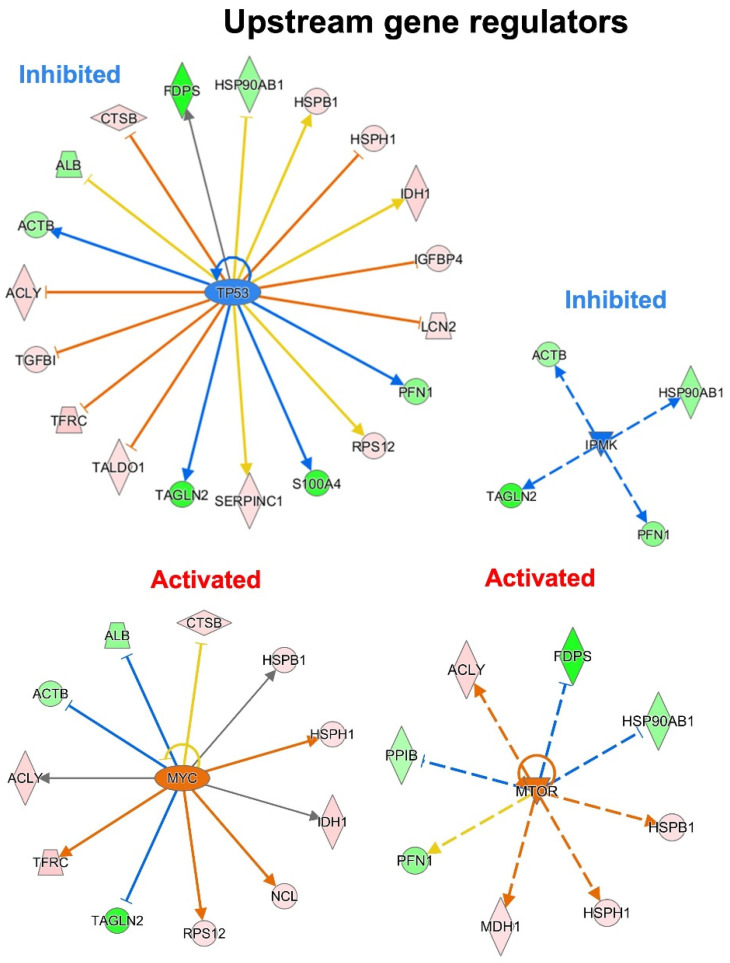
Upstream gene regulator analysis. In p53 knocking-down conditions, TP53 and IPMK are the most significant inhibited upstream regulators, while MYC and MTOR resulted the most significant activated regulators.

To predict the upstream molecules (genes, transcription factors, microRNA, etc.) that may play a role in the observed secretome modulation and, thus, in response to a p53 silencing in PDAC, we performed the upstream regulator analysis through IPA software. IPA analysis suggested that TP53, IPMK, MYC and MTOR are among the most significant upstream regulators predicted as inhibited or activated (Figure 6). Interestingly, the most significant upstream regulator, with a *p*-value of 2.57^−10^, was TP53. TP53 was predicted inhibited (z-score of −1.289) and the 40.4% of the total modulated proteins resulted under its regulation. Among these, eight proteins that are inhibited by TP53 were detected as hypersecreted (ACLY, TGFB1, TFRC, TALDO1, LCN2, IGFBP4, HSPH1, CTSB) after p53 knock-down, whereas five proteins that are activated by TP53 resulted to be hyposecreted (ACTB, TAGLN2, S100A4, PFN1, FDPS) after the p53 knock-down in PaCa3 cells. Moreover, the upstream regulators MYC and MTOR were predicted as activated (z-score of 2.157 and 2.984, respectively) with a *p*-value of 2.75^−7^ and 1.17^−6^, respectively. Instead, the upstream regulator IPMK was predicted as inhibited (z-score of −2.00) with a *p*-value of 9.87^−8^. MYC and MTOR are well-known oncogenes usually activated in cancer, whereas IPMK can act as a co-activator of TP53 [29,30].

**Figure 7:**
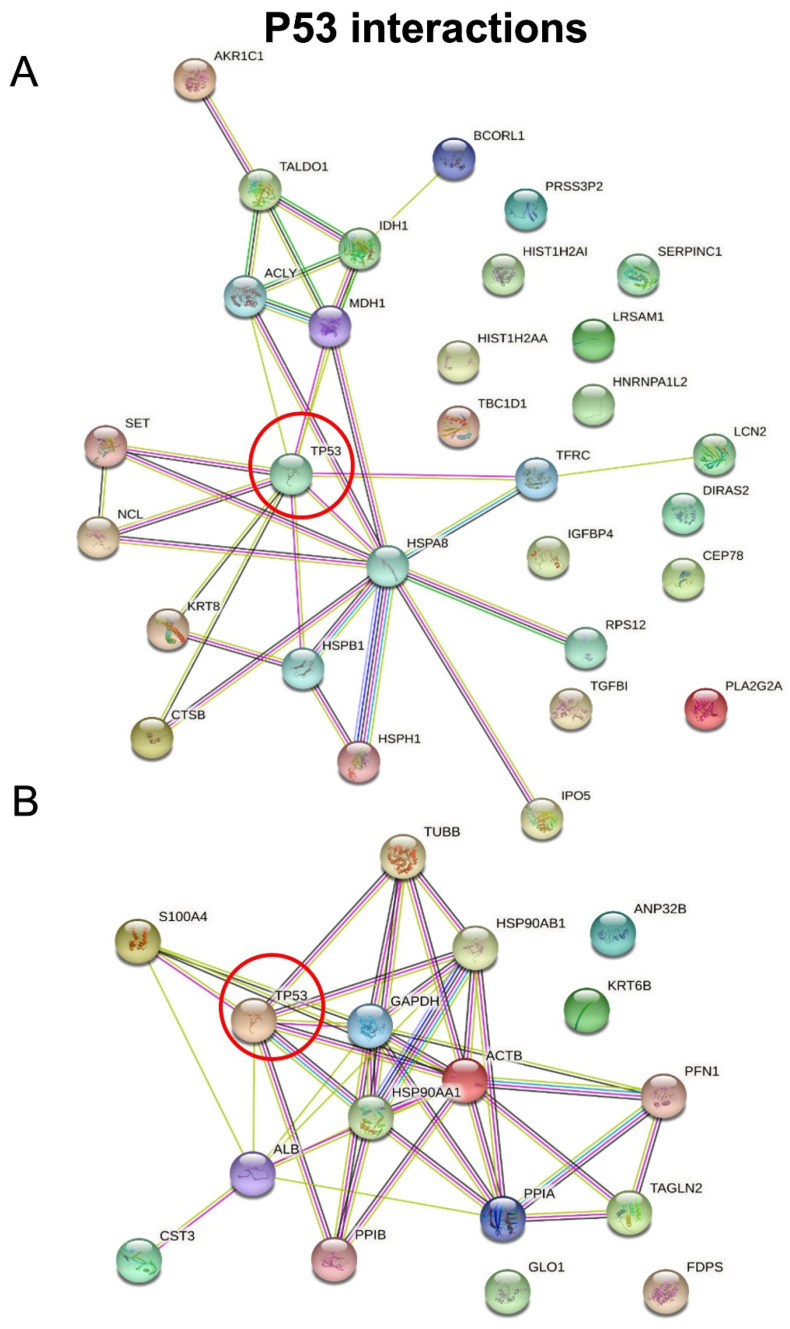
STRING analysis. Protein–protein interactions among modulated proteins. p53 was manually added to identify potentially related connections. The network of hypersecreted proteins is reported in (**A**) while the network of hyposecreted proteins is reported in (**B**).

We then employed STRING software in order to investigate protein–protein interactions and to explore the functional enriched pathways. We manually added p53 to the list of regulated proteins and found that it directly interacts with nine hypersecreted (Figure 7A) and eight hyposecreted proteins (Figure 7B). Concerning the hypersecreted proteins after p53 knock-down, the network analysis reported a cluster of interaction between p53 and SET, HSPB1, K2C8, NUCL, CATB, TFR1, MDHC, HSP7C, ACLY; while among the hyposecreted proteins after p53 knock-down there was a cluster of interaction involving S10A4, ALBU, PPIB, HS90B, HS90A, ACTB, G3P, TBB5. The functional enrichment analysis also showed that most of the hypersecreted proteins belong to extracellular space and exosomes, supporting their role in cell–cell communications.

Finally, the diseases and functions analysis performed through the IPA software highlighted the involvement of proteins associated with leukocytes movement (HSPB1, PPIB, ACTB, HS90B, ALB and S10A4), carcinoma (CATB, ALB, CYTC and SAA4) and advanced malignant tumor (CATB, BGH3, ACTB, CYTC and SAA4) disease classes (Table 2).

## 4. Discussion and Conclusions

The p53 tumor suppressor protein is a key controller of tumor development. Intrinsic and extrinsic stress signals are transferred to the p53 protein by post-translational modifications promoting p53 protein activation [31,32]. The activated p53 acts as a transcription factor triggering several biological phenomena, including cell cycle arrest, cellular senescence or apoptosis. The p53-responsive gene transcriptional network results in proteins that interact with a large number of other signal transduction pathways in the cell, and a number of self-regulatory feedback loops act on the p53 response [32]. In addition to the well-known canonical pathways of p53, it also induces non-canonical pathways [33]. Indeed, the importance of p53 target gene spectrum is also reflected in the tumor microenvironment through the regulation of numerous genes encoding for secreted proteins that trigger changes outside of the tumor cell [34,35]. Upon various cancer therapy agents, the hepatic-activated p53 induces the secretion of proteins involved in hemostasis, immune response and cell adhesion [35]. Furthermore, p53 loss activates pro-tumorigenic secretory pathway and Golgi reprogramming is a driver of hypersecretion in cancer [36,37,38]. Thus, a loss of p53 has a profound impact on the secretome composition of cancer cells and marks the transition to invasiveness. Here, we discovered the functional tumor suppressor role of wild-type p53 driven secretome, which is able to counteract cell growth and chemoresistance and promote cell death-related mechanisms in pancreatic adenocarcinoma cells, in accordance with the tumor suppressor role of p53 protein. Thus, by using high-resolution SWATH-MS technology, we characterized the secretomes of p53-deficient and p53-expressing PaCa3 PDAC cells. We identified 30 hypersecreted proteins and 17 hyposecreted proteins in response to p53 silencing in PDAC. The bioinformatic analysis allowed the identification of the main canonical pathways associated with the silencing of p53. Aldosterone signaling in epithelial cells, which is related in our data to the modulation of the chaperones proteins heat shock protein beta-1, heat shock-related 70 kDa protein 2, heat shock protein 105 kDa and heat shock protein HSP 90-beta, resulted in the most significant modulated pathway. Aldosterone exhibits direct effect on the epithelial cells and it is able to activate inflammation via p38MAPK and NF-kB [39]: for this reason, an involvement of this pathway in the cancerous secretome can be linked to the intense inflammation typical of tumor microenvironment. Despite mineralocorticoids as aldosterone, glucocorticoids are also involved in the pathways underlined by bioinformatic analysis: indeed, the modulated proteins actin 1, heat shock protein HSP 90-beta, heat shock-related 70 kDa protein 2, KRT6A and PLA2G2A were involved in the glucocorticoid receptor signaling. It is reported that in human pancreatic carcinoma there is a strong expression of these receptors: their carcinogenic effect is probably related to their capability of binding the oncogenes c-Myc, c-Jun and NF-kB [40]. An important role of the cellular metabolism emerged from our data: proteins related to gluconeogenesis (GAPDH5 and MDH1) and acetyl-CoA biosynthesis (ACLY) provoked modulation in the secretome. Gluconeogenesis is essential for cancer growth because it supplies the nutrients necessary for the continuous proliferation of the cancer cells through amino acids and lactate [41]. In addition, to support the constant need of nutrients by the cancer cells, the acetyl-CoA synthesis and the synthesis of fatty acids are crucial. Moreover, it has been described that fatty acids are able to avoid apoptosis in cancer cells [42]. Another interesting pathway that has emerged is the protein ubiquitination pathway, which comprises the chaperones HSP90AB1, HSPA2, HSPB1 and HSPH1: it is well-known that oncogenic/oncosuppressor proteins are also modulated by the ubiquitin-proteasome system, whose main actors are chaperone proteins [43]. The pathways enrichment analysis highlighted the implication of secreted proteins in metastasis formation. Clathrin-mediated endocytosis signaling involves the regulated proteins ACTB, ALB and TRFC: this kind of endocytosis is related to the traffic of cell-secreted vesicles, exosomes and to the communication between neighboring cells, which are key events in cancer, especially for the spread of metastasis [44]. ACTB and TUBB2A participate in the remodeling of epithelial adherens junctions, which is another pathway with a role in the metastasis formation and in the epithelial to mesenchymal transition: in fact, the adherens junctions are essential for the maintenance of the shape, polarity and functionality of the epithelial cell, through the E-cadherin–catenin complex. The loss of these junctions is a crucial step for the epithelial to mesenchymal transition, which leads to the diffusion of metastases [45].

Proteins associated with cell death and survival were also identified through the molecular and cellular functions analysis. It is extremely clear that impairment in the apoptosis pathways and immortalization of cells are events at the basis of cancerogenesis [46]. Another perturbed function was the protein synthesis, which is usually widely perturbed in malignancies, leading to an impaired differentiation of the cell and to a failed synthesis of important proteins [47]. In addition, an altered cellular movement is crucial for the onset of cancer: the malignant cells can migrate in tissue, following the degradation of the extracellular matrix, setting the stage for metastasis formation [48].

Among modulated proteins, we identified an upregulation of BCORL (BCL-6 corepressor-like protein 1) protein. In literature it is reported that BCORL has an oncogenic role. More precisely, BCORL is a corepressor of the BCL6 repressor protein, so it acts together with BCL6 in its transcriptional repressive activity which leads to the block of apoptosis [49,50]. Moreover, Pagan et al. reported that BCORL can repress E-cadherin thanks to an interaction with CtBP, which transcriptionally regulates the expression of E-cadherin [50]. It has been widely described that the loss of E-cadherin, which is a protein responsible of the epithelial adhesion, is the key event for the loss of cell–cell adhesion that is one of the crucial steps for the dissemination of metastasis [51]. We can then speculate that the silencing of p53 or its impairment can cause an overexpression of BCORL, suggesting that its secretion is repressed in the presence of WTp53. This mechanism seems to be really intriguing: in fact, the transcription repressor BCORL might be transmitted from a cancerous cell to another, after its secretion in the extracellular microenvironment, contributing to create a pro-metastatic secretome.

Furthermore, ingenuity pathway analysis reported that p53 is an upstream regulator for 19 of the 47 modulated proteins. For all the eight proteins inhibited by p53 and thus hypersecreted in the p53-silenced secretome, an oncogenic role has emerged from literature. ACLY (ATP-citrate synthase) is an enzyme which takes part in the conversion of glucose into lipid species, which are used by the cancer cells to create new lipid membranes to accelerate the proliferation, as already reported in pancreatic cancer [52]. In PDAC cells, lipid species are also important for the synthesis of signaling molecules and to provide molecules for protein post-translational modifications [53]. ACLY, together with the other two hypersecreted proteins MDHC and IDHC, is also involved in cell metabolism, and in particular in the TCA cycle, which is used by the cell to produce energy and to synthesize macromolecules. It has been recently observed that TCA cycle is up-regulated in cancer cells that show a deregulation of tumour suppressor and oncogenes [54]. Despite the cell metabolism, there are other molecular functions that leads to proliferation-inducing effect, and which are carried out by secreted proteins caused by the inhibition of p53. As an example, TFR1 (Transferrin receptor protein 1) has been already associated with cell proliferation in PDAC. Indeed, a huge quantity of TFR1 improves oxidative phosphorylation, that leads to the generation of ROS, which are crucial for pancreatic adenocarcinoma growth [55]. In addition, it has been reported that TFR1 is usually overexpressed in pancreatic cancer: in fact, it is considered a marker of malignant transformation [56]. Another hypersecreted protein involved in proliferation is NGAL (Neutrophil gelatinase-associated lipocalin), highly up-regulated in pancreatic cancer cells [57] and identified in the secretome of thyroid neoplastic cells as a factor implicated in the survival [58]. In addition, the hypersecreted IBP4 (Insulin-like growth factor-binding protein 4) has a role in cell proliferation and tumour growth, and its over-secretion has been already reported by pancreatic cancer cells [59]. HSPH1 (heat shock protein 105 kDa), another hypersecreted protein after p53 knock-down, was found over-secreted in pancreatic cancer. This protein is involved in protein folding, cellular process usually perturbed in cancer [59]. The hypersecreted proteins CATB, TALDO1 and BGH3 are involved in another key feature of the cancer aggressiveness: the process of metastasis and invasion. CATB (Cathepsin B) is a protein belonging to the Hedgehog pathway and an intense signaling of this protein has been associated with an increase in the invasive properties of the pancreatic tumor [60]. It is amply reported that cathepsin is usually secreted by cells of the cancerous microenvironment and it has a role in the formation of metastasis [60]. Moreover, TALDO1 overexpression is associated with an increased tumor size and presence of metastases in several cancers [61]. However, its molecular mode of action in this context is not fully understood. Since in Bifidobacterium it is involved in cell aggregation and mucin adhesion in the extracellular compartment, Brandi et al. have speculated that in pancreatic cancer cells secretome, TALDO1 enhances invasiveness and metastasis acting on mucins [62]. Finally, BGH3 (Transforming growth factor-beta-induced protein ig-h3) has been found up-regulated in many cancers, including pancreatic cancer, and there is evidence of its role in metastasis formation in colorectal and liver cancers. Moreover, the secretion of BGH3 by pancreas cancerous cells can induce proliferation of the stellate cells of pancreas [62]. Ingenuity pathway analysis reported that p53 is also an upstream regulator that down-regulates a panel of secreted proteins. Concerning these proteins, a dual role has emerged for most of them, both antitumorigenic and tumorigenic. TAGLN2 (Transgelin 2) is essential for the host defense against cancer and infections, and it is secreted by activated macrophages. It binds to actin, modulating the activation of T-cells by the actin-mediated stabilization of T-cell cytoskeleton, immunological synapsis and B-cell–T-cell conjugates [62]. However, it has been also reported that its expression is important for the development of some types of tumors [62] by the activation of PI3K/Akt/GSK-3beta pathway, increasing migration and invasiveness [63]. ACTB (Actin cytoplasmic 1) was down-regulated in the inflammatory microenvironment of hepatocellular carcinoma [63]. Another under-expressed protein is S10A4: it could be involved in the metastasis formation, but it has also been observed in immune cells with antitumor activity. For example, this protein is present on the surface of CD4+ T-cells and it leads to the recognition of the tumor cells, provoking their lysis [63]. On the other hand, in the extracellular space S10A4 can be implicated in the sensitization of tumor cells to IFN-gamma-mediated apoptosis. Interestingly, an interaction and a cross-talk between S100A4 and p53, which might be related to its oncosuppressive properties, has been already reported [64]. In fact, it has been observed that the concentration of S10A4 decreases in cancer cells with the silencing of p53 [65]. Globally, the down-regulation of these proteins underlines an impairment of the functionality of immune cells in the tumor microenvironment. In support of this, ingenuity pathway analysis has reported the detriment of the “Leukocytes movement” function, as a consequence of the hypersecretion of HSPB1 and the hyposecretion of PPIB, ACTB, HS90B, ALB and S10A4. It has been widely described that the escape of the cancer cells from the anticancer immune response is a fundamental event for cancer onset and development. It has been suggested that the oncosuppressor p53 can modulate the response of the immune cells against cancer: indeed, the silencing of p53 provokes an impairment of the T-helper and T-cytotoxic action and promotes the differentiation of macrophages into the oncogenic M2 phenotype. Conversely, the expression of p53 leads to a tumoral immune response in the cancer stroma [66].

As predicted, the bioinformatic analysis has also reported activated up-stream regulators of the transcription factor c-Myc and the kinase mTOR, whereas the kinase IPMK was predicted as inhibited. c-Myc is a well-known oncogenic protein involved in cell growth, whose over-regulation has been widely detected in cancer [67]. In addition, a relation between the c-Myc expression and the inactivation of p53 has been demonstrated, even if the molecular basis of this relation has not been demonstrated yet [68]. Among the up-regulated proteins activated by c-Myc, there is RPS1, which is related to an increase in the cell motility and of the invasive capabilities of cancer cells [69] and nucleolin, whose expression is usually perturbed in cancer cells and promotes cancer progression [70]. Another protein activated by c-Myc is HSPH1, which is responsible for the destruction of Beta-catenin and is essential for cell–cell adhesion and whose reduction is associated with cancerous transformation [70]. The up-stream regulator mTOR is a widely described oncogene, frequently overexpressed in many neoplasms [71]. It activates the upstream regulated MDH1 enzyme, which is reported to be activated in pancreatic cancer, promoting its growth [72]. Another interesting up-regulated protein activated by mTOR is HSPB1, which is a chaperone protein frequently detected in cancer tissues that leads to a poor survival of the oncologic patients by causing an increase in cancer proliferation and by down-regulating the anticancer immune action. Finally, the IPMK kinase was predicted as an inhibited upstream regulator. IPMK is strictly involved in autophagy, which is a molecular mechanism fundamental for the preservation of the cell homeostasis [73]. Autophagy is often described as a mechanism of survival for the cell; however, it has been observed that chemotherapy can provoke a kind of autophagy that causes cell death [74].

In conclusion, although the suppressor role of p53-driven conditioned medium may also depend on other differentially secreted molecules, as for instance RNAs or others, the present study allowed a strong advancement on our understanding of the link between WTp53 and the cancer microenvironment, suggesting the presence of a specific proteomic signature driven by WTp53 in PDAC.

## Figures and Tables

**Table 1:** Forty-seven modulated proteins in PDAC secretome of knock-down p53 Paca3 cells, as compared to wild-type p53 cells identified by SWATH-MS technology (*p* < 0.05). Fold change (FC) represents the ratio between CM protein abundance of p53 knock-down (KD) and p53 wild-type PaCa3 cells.

Uniprot ID	Uniprot Accession Name	Protein Name	Gene Name	FC (KD/wt p53)	*p*-Value
O00410	IPO5_HUMAN	Importin-5	IPO5	4.7	4.31 × 10^−4^
O75874	IDHC_HUMAN	Isocitrate dehydrogenase	IDH1	3.0	3.56 × 10^−4^
P01008	ANT3_HUMAN	Antithrombin-III	SERPINC1	1.6	2.66 × 10^−4^
P02786	TFR1_HUMAN	Transferrin receptor protein 1	TFRC	3.8	8.59 × 10^−4^
P04792	HSPB1_HUMAN	Heat shock protein beta-1	HSPB1	1.8	1.72 × 10^−2^
P04908	H2A1_HUMAN	Histone H2A type 1-B/E	HIST1	2.1	2.39 × 10^−3^
P07478	TRY2_HUMAN	Trypsin-2	PRSS2	1.3	4.27 × 10^−2^
P07858	CATB_HUMAN	Cathepsin B	CTSB	2.6	3.33 × 10^−3^
P14136	K2C8_HUMAN	Glial fibrillary acidic protein	GFAP	5.5	1.49 × 10^−2^
P14555	PA2GA_HUMAN	Phospholipase A2	PLA2G2A	2.4	1.86 × 10^−4^
P19338	NUCL_HUMAN	Nucleolin	NCL	1.4	9.66 × 10^−4^
P22692	IBP4_HUMAN	Insulin-like growth factor-binding protein 4	IGFBP4	1.9	9.48 × 10^−3^
P25398	RS12_HUMAN	40S ribosomal protein S12	RPS12	1.6	2.92 × 10^−2^
P37837	TALDO_HUMAN	Transaldolase	TALDO1	1.4	4.35 × 10^−3^
P40925	MDHC_HUMAN	Malate dehydrogenase	MDH1	1.8	9.26 × 10^−3^
P53396	ACLY_HUMAN	ATP-citrate synthase	ACLY	2.9	6.18 × 10^−4^
P54652	HSP7C_HUMAN	Heat shock-related 70 kDa protein 2	HSPA2	1.7	6.79 × 10^−3^
P62158	CALM_HUMAN	Calmodulin	CALM1	1.5	1.31 × 10^−2^
P80188	NGAL_HUMAN	Neutrophil gelatinase-associated lipocalin	LCN2	2.0	7.20 × 10^−5^
Q01105	SET_HUMAN	Protein SET	SET	1.6	1.55 × 10^−2^
Q04828	AK1C1_HUMAN	Aldo-keto reductase family 1 member C1	AKR1C1	1.5	8.20 × 10^−3^
Q15582	BGH3_HUMAN	Transforming growth factor-beta-induced protein ig-h3	TGFBI	1.4	5.93 × 10^−3^
Q32P51	RA1L2_HUMAN	Heterogeneous nuclear ribonucleoprotein A1-like 2	HNRNPA1L2	1.3	7.76 × 10^−3^
Q5H9F3	BCORL_HUMAN	BCL-6 corepressor-like protein 1	BCORL1	24.3	5.74 × 10^−5^
Q6UWE0	LRSM1_HUMAN	E3 ubiquitin-protein ligase LRSAM1	LRSAM1	2.2	6.48 × 10^−3^
Q86TI0	TBCD1_HUMAN	TBC1 domain family member 1	TBC1D1	2.5	8.13 × 10^−5^
Q92598	HS105_HUMAN	Heat shock protein 105 kDa	HSPH1	1.9	1.65 × 10^−2^
Q96HU8	DIRA2_HUMAN	GTP-binding protein Di-Ras2	DIRAS2	36.4	4.02 × 10^−4^
Q96QV6	H2A1A_HUMAN	Histone H2A type 1-A	HIST1H2AA	1.3	1.29 × 10^−3^
A8MST6	CEP78_HUMAN	Centrosomal protein of 78 kDa	CEP78	23.7	2.99 × 10^−3^
B2RPK0	HGB1A_HUMAN	Putative high mobility group protein B1-like 1	HMGB1P1	0.6	8.43 × 10^−3^
O14556	G3P_HUMAN	Glyceraldehyde-3-phosphate dehydrogenase	GAPDHS	0.5	2.66 × 10^−2^
P01034	CYTC_HUMAN	Cystatin-C	CST3	0.6	4.58 × 10^−3^
P02538	K2C6B_HUMAN	Keratin, type II cytoskeletal 6A	KRT6A	0.5	1.53 × 10^−3^
P02768	ALBU_HUMAN	Serum albumin	ALB	0.6	4.06 × 10^−4^
P07737	PROF1_HUMAN	Profilin-1	PFN1	0.5	9.28 × 10^−6^
P08238	HS90B_HUMAN	Heat shock protein HSP 90-beta	HSP90AB1	0.6	1.14 × 10^−2^
P14324	FPPS_HUMAN	Farnesyl pyrophosphate synthase	FDPS	0.3	9.93 × 10^−4^
P23284	PPIB_HUMAN	Peptidyl-prolyl cis-trans isomerase B	PPIB	0.7	1.40 × 10^−2^
P26447	S10A4_HUMAN	Protein S100-A4	S100A4	0.3	1.72 × 10^−4^
P37802	TAGL2_HUMAN	Transgelin-2	TAGLN2	0.3	2.18 × 10^−3^
P60709	ACTB_HUMAN	Actin, cytoplasmic 1	ACTB	0.6	5.29 × 10^−5^
Q04760	LGUL_HUMAN	Lactoylglutathione lyase	GLO1	0.5	5.79 × 10^−4^
Q13885	TBB5_HUMAN	Tubulin beta-3 chain	TUBB3	0.3	2.70 × 10^−3^
Q58FG1	HS90A_HUMAN	Putative heat shock protein HSP 90-alpha A4	HSP90AA4P	0.3	2.16 × 10^−3^
Q92688	AN32B_HUMAN	Acidic leucine-rich nuclear phosphoprotein 32 family member B	ANP32B	0.4	5.73 × 10^−3^
Q9Y536	PPIA_HUMAN	Peptidyl-prolyl cis-trans isomerase A-like 4A	PPIAL4A	0.4	3.52 × 10^−3^

**Table 2:** Diseases and functions analysis. Leukocytes movement, carcinoma and advanced malignant tumor resulted the most significant diseases and functions classes associated with modulated proteins. The proteins with their function and regulation after p53 knock-down are reported in the table.

**Diseases and Functions**		
***Leukocytes movement***		
**Protein**	**Regulation**	**Function**
HSPB1	Up-regulated	Decrease movement of leucocytes
PPIB	Down-regulated	Increase movement of leucocytes
ACTB	Down-regulated	Increase movement of leucocytes
HS90B	Down-regulated	Increase movement of leucocytes
ALB	Down-regulated	Increase movement of leucocytes
S10A4	Down-regulated	Increase movement of leucocytes
***Carcinoma***		
**Protein**	**Regulation**	**Function**
CATB	Up-regulated	Increase carcinoma
ALB	Down-regulated	Decrease carcinoma
CYTC	Down-regulated	Decrease carcinoma
SAA4	Down-regulated	Decrease carcinoma
***Advanced malignant tumour***		
**Protein**	**Regulation**	**Function**
CATB	Up-regulated	Increase advanced malignant tumour
BGH3	Up-regulated	Increase advanced malignant tumour
ACTB	Down-regulated	Decrease advanced malignant tumour
CYTC	Down-regulated	Decrease advanced malignant tumour
SAA4	Down-regulated	Increase advanced malignant tumour

## Data Availability

Not applicable.

## References

[1] Adamska A., Domenichini A., Falasca M. (2017). Pancreatic Ductal Adenocarcinoma: Current and Evolving Therapies. Int. J. Mol. Sci..

[2] Smith J.K., Chu Q.D., Tseng J.F. (2015). Pancreatic Adenocarcinoma. Surgical Oncology.

[3] Ducreux M., Cuhna A.S., Caramella C., Hollebecque A., Burtin P., Goéré D., Seufferlein T., Haustermans K., Van Laethem J.L., Conroy T. (2015). Cancer of the pancreas: ESMO Clinical Practice Guidelines for diagnosis, treatment and follow-up. Ann. Oncol..

[4] Kastan M.B. (2007). Wild-Type p53: Tumors Can’t Stand It. Cell.

[5] Kruse J.-P., Gu W. (2009). Modes of p53 Regulation. Cell.

[6] Vousden K.H., Prives C. (2009). Blinded by the Light: The Growing Complexity of p53. Cell.

[7] Strano S., Dell’Orso S., Di Agostino S., Fontemaggi G., Sacchi A., Blandino G. (2007). Mutant p53: An oncogenic transcription factor. Oncogene.

[8] Di Marco M., Astolfi A., Grassi E., Vecchiarelli S., Macchini M., Indio V., Casadei R., Ricci C., D’Ambra M., Taffurelli G. (2015). Characterization of pancreatic ductal adenocarcinoma using whole transcriptome sequencing and copy number analysis by single-nucleotide polymorphism array. Mol. Med. Rep..

[9] Hollstein M., Sidransky D., Vogelstein B., Harris C.C. (1991). p53 Mutations in Human Cancers. Science.

[10] Freed-Pastor W.A., Prives C. (2012). Mutant p53: One name, many proteins. Genes Dev..

[11] Cho S.-Y., Park C., Na D., Han J.Y., Lee J., Park O.-K., Zhang C., Sung C.O., Moon H.E., Kim Y. (2017). High prevalence of TP53 mutations is associated with poor survival and an EMT signature in gliosarcoma patients. Exp. Mol. Med..

[12] Blandino G., Levine A.J., Oren M. (1999). Mutant p53 gain of function: Differential effects of different p53 mutants on resistance of cultured cells to chemotherapy. Oncogene.

[13] Butera G., Pacchiana R., Mullappilly N., Margiotta M., Bruno S., Conti P., Riganti C., Donadelli M. (2018). Mutant p53 prevents GAPDH nuclear translocation in pancreatic cancer cells favoring glycolysis and 2-deoxyglucose sensitivity. Biochim. Biophys. Acta Mol. Cell Res..

[14] Cordani M., Butera G., Dando I., Torrens-Mas M., Butturini E., Pacchiana R., Oppici E., Cavallini C., Gasperini S., Tamassia N. (2018). Mutant p53 blocks SESN1/AMPK/PGC-1α/UCP2 axis increasing mitochondrial O2ˉ· production in cancer cells. Br. J. Cancer.

[15] Fiorini C., Cordani M., Padroni C., Blandino G., Di Agostino S., Donadelli M. (2015). Mutant p53 stimulates chemoresistance of pancreatic adenocarcinoma cells to gemcitabine. Biochim. Biophys. Acta Mol. Cell Res..

[16] Butera G., Brandi J., Cavallini C., Scarpa A., Lawlor R.T., Scupoli M.T., Marengo E., Cecconi D., Manfredi M., Donadelli M. (2020). The Mutant p53-Driven Secretome Has Oncogenic Functions in Pancreatic Ductal Adenocarcinoma Cells. Biomolecules.

[17] Cordani M., Pacchiana R., Butera G., D’Orazi G., Scarpa A., Donadelli M. (2016). Mutant p53 proteins alter cancer cell secretome and tumour microenvironment: Involvement in cancer invasion and metastasis. Cancer Lett..

[18] Brandi J., Manfredi M., Speziali G., Gosetti F., Marengo E., Cecconi D. (2018). Proteomic approaches to decipher cancer cell secretome. Semin. Cell Dev. Biol..

[19] Manfredi M., Martinotti S., Gosetti F., Ranzato E., Marengo E. (2016). The secretome signature of malignant mesothelioma cell lines. J. Proteom..

[20] Albanese P., Manfredi M., Re A., Marengo E., Saracco G., Pagliano C. (2018). Thylakoid proteome modulation in pea plants grown at different irradiances: Quantitative proteomic profiling in a non-model organism aided by transcriptomic data integration. Plant J..

[21] MacLean B., Tomazela D.M., Shulman N., Chambers M., Finney G.L., Frewen B., Kern R., Tabb D.L., Liebler D.C., MacCoss M.J. (2010). Skyline: An open source document editor for creating and analyzing targeted proteomics experiments. Bioinformatics.

[22] Manfredi M., Brandi J., Di Carlo C., Vita Vanella V., Barberis E., Marengo E., Patrone M., Cecconi D. (2019). Marengo Mining cancer biology through bioinformatic analysis of proteomic data. Expert Rev. Proteom..

[23] Szklarczyk D., Gable A.L., Lyon D., Junge A., Wyder S., Huerta-Cepas J., Simonovic M., Doncheva N.T., Morris J.H., Bork P. (2019). STRING v11: Protein–protein association networks with increased coverage, supporting functional discovery in genome-wide experimental datasets. Nucleic Acids Res..

[24] Tasdemir E., Maiuri M.C., Galluzzi L., Vitale I., Djavaheri-Mergny M., D’Amelio M., Criollo A., Morselli E., Zhu C., Harper F. (2008). Regulation of autophagy by cytoplasmic p53. Nat. Cell Biol..

[25] Cordani M., Butera G., Pacchiana R., Donadelli M. (2017). Molecular interplay between mutant p53 proteins and autophagy in cancer cells. Biochim. Biophys. Acta Rev. Cancer.

[26] El-Deiry W.S. (2003). The role of p53 in chemosensitivity and radiosensitivity. Oncogene.

[27] Baker S.J., Markowitz S., Fearon E.R., Willson J.K.V., Vogelstein B. (1990). Suppression of Human Colorectal Carcinoma Cell Growth by Wild-Type p53. Science.

[28] Shaw P., Bovey R., Tardy S., Sahli R., Sordat B., Costa J. (1992). Induction of apoptosis by wild-type p53 in a human colon tumor-derived cell line. Proc. Natl. Acad. Sci. USA.

[29] Pópulo H., Lopes J.M., Soares P. (2012). The mTOR Signalling Pathway in Human Cancer. Int. J. Mol. Sci..

[30] Dong Y., Tu R., Liu H., Qing G. (2020). Regulation of cancer cell metabolism: Oncogenic MYC in the driver’s seat. Signal Transduct. Target. Ther..

[31] Kruiswijk F., Labuschagne C.F., Vousden K.H. (2015). p53 in survival, death and metabolic health: A lifeguard with a licence to kill. Nat. Rev. Mol. Cell Biol..

[32] Harris S.L., Levine A.J. (2005). The p53 pathway: Positive and negative feedback loops. Oncogene.

[33] Bieging K.T., Mello S.S., Attardi L.D. (2014). Unravelling mechanisms of p53-mediated tumour suppression. Nat. Rev. Cancer.

[34] Pavlakis E., Stiewe T. (2020). p53′s Extended Reach: The Mutant p53 Secretome. Biomolecules.

[35] Charni-Natan M., Solomon H., Molchadsky A., Jacob-Berger A., Goldfinger N., Rotter V. (2018). Various stress stimuli rewire the profile of liver secretome in a p53-dependent manner. Cell Death Dis..

[36] Tan X., Banerjee P., Shi L., Xiao G.-Y., Rodriguez B.L., Grzeskowiak C.L., Liu X., Yu J., Gibbons D.L., Russell W.K. (2021). p53 loss activates prometastatic secretory vesicle biogenesis in the Golgi. Sci. Adv..

[37] Tan X., Shi L., Banerjee P., Liu X., Guo H.-F., Yu J., Bota-Rabassedas N., Rodriguez B.L., Gibbons D.L., Russell W.K. (2021). A protumorigenic secretory pathway activated by p53 deficiency in lung adenocarcinoma. J. Clin. Investig..

[38] Khwaja F.W., Svoboda P., Reed M., Pohl J., Pyrzynska B., Van Meir E.G. (2006). Proteomic identification of the wt-p53-regulated tumor cell secretome. Oncogene.

[39] Zhu C.-J., Wang Q.-Q., Zhou J.-L., Liu H.-Z., Hua F., Yang H.-Z., Hu Z.-W. (2012). The mineralocorticoid receptor-p38MAPK-NFκB or ERK-Sp1 signal pathways mediate aldosterone-stimulated inflammatory and profibrotic responses in rat vascular smooth muscle cells. Acta Pharmacol. Sin..

[40] Bekasi S., Zalatnai A. (2009). Overexpression of Glucocorticoid Receptor in Human Pancreatic Cancer and in Xenografts. An Immunohistochemical Study. Pathol. Oncol. Res..

[41] Grasmann G., Smolle E., Olschewski H., Leithner K. (2019). Gluconeogenesis in cancer cells—Repurposing of a starvation-induced metabolic pathway? Biochim. Biophys. Acta Rev. Cancer.

[42] Yoshii Y., Furukawa T., Saga T., Fujibayashi Y. (2015). Acetate/acetyl-CoA metabolism associated with cancer fatty acid synthesis: Overview and application. Cancer Lett..

[43] Burger A.M., Seth A.K. (2004). The ubiquitin-mediated protein degradation pathway in cancer: Therapeutic implications. Eur. J. Cancer.

[44] Casari I., Howard J.A., Robless E.E., Falasca M. (2021). Exosomal integrins and their influence on pancreatic cancer progression and metastasis. Cancer Lett..

[45] Baum B., Georgiou M. (2011). Dynamics of adherens junctions in epithelial establishment, maintenance, and remodeling. J. Cell Biol..

[46] Jäättelä M. (1999). Escaping Cell Death: Survival Proteins in Cancer. Exp. Cell Res..

[47] Hershey J.W. (2010). Regulation of protein synthesis and the role of eIF3 in cancer. Braz. J. Med. Biol. Res..

[48] Paul C.D., Mistriotis P., Konstantopoulos K. (2017). Cancer cell motility: Lessons from migration in confined spaces. Nat. Rev. Cancer.

[49] Kurosu T., Fukuda T., Miki T., Miura O. (2003). BCL6 overexpression prevents increase in reactive oxygen species and inhibits apoptosis induced by chemotherapeutic reagents in B-cell lymphoma cells. Oncogene.

[50] Pagan J.K., Arnold J., Hanchard K.J., Kumar R., Bruno T., Jones M.J.K., Richard D.J., Forrest A., Spurdle A., Verdin E. (2007). A Novel Corepressor, BCoR-L1, Represses Transcription through an Interaction with CtBP. J. Biol. Chem..

[51] Onder T.T., Gupta P.B., Mani S.A., Yang J., Lander E.S., Weinberg R.A. (2008). Loss of E-Cadherin Promotes Metastasis via Multiple Downstream Transcriptional Pathways. Cancer Res..

[52] Schlichtholz B., Turyn J., Goyke E., Biernacki M., Jaskiewicz K., Sledzinski Z., Swierczynski J. (2005). Enhanced Citrate Synthase Activity in Human Pancreatic Cancer. Pancreas.

[53] Qin C., Yang G., Yang J., Ren B., Wang H., Chen G., Zhao F., You L., Wang W., Zhao Y. (2020). Metabolism of pancreatic cancer: Paving the way to better anticancer strategies. Mol. Cancer.

[54] Anderson N.M., Mucka P., Kern J.G., Feng H. (2018). The emerging role and targetability of the TCA cycle in cancer metabolism. Protein Cell.

[55] Jeong S.M., Hwang S., Seong R. (2016). Transferrin receptor regulates pancreatic cancer growth by modulating mitochondrial respiration and ROS generation. Biochem. Biophys. Res. Commun..

[56] Ryschich E., Huszty G., Knaebel H., Hartel M., Büchler M., Schmidt J. (2004). Transferrin receptor is a marker of malignant phenotype in human pancreatic cancer and in neuroendocrine carcinoma of the pancreas. Eur. J. Cancer.

[57] Moniaux N., Chakraborty S., Yalniz M., Gonzalez J., Shostrom V.K., Standop J., Lele S.M., Ouellette M., Pour P.M., Sasson A.R. (2008). Early diagnosis of pancreatic cancer: Neutrophil gelatinase-associated lipocalin as a marker of pancreatic intraepithelial neoplasia. Br. J. Cancer.

[58] Karagiannis G.S., Pavlou M.P., Diamandis E.P. (2010). Cancer secretomics reveal pathophysiological pathways in cancer molecular oncology. Mol. Oncol..

[59] Schiarea S., Solinas G., Allavena P., Scigliuolo G.M., Bagnati R., Fanelli R., Chiabrando C. (2010). Secretome Analysis of Multiple Pancreatic Cancer Cell Lines Reveals Perturbations of Key Functional Networks. J. Proteome Res..

[60] Hwang J.-H., Lee S.H., Lee K.H., Lee K.Y., Kim H., Ryu J.K., Yoon Y.B., Kim Y.-T. (2009). Cathepsin B is a target of Hedgehog signaling in pancreatic cancer. Cancer Lett..

[61] Wu Y., Lee Y., Li W., Hsu W., Lin H., Chang L., Huang A., Jhan J., Wu W., Li C. (2021). High Transaldolase 1 expression predicts poor survival of patients with upper tract urothelial carcinoma. Pathol. Int..

[62] Brandi J., Pozza E.D., Dando I., Biondani G., Robotti E., Jenkins R., Elliott V., Park K., Marengo E., Costello E. (2016). Secretome protein signature of human pancreatic cancer stem-like cells. J. Proteom..

[63] Liu L., Meng T., Zheng X., Liu Y., Hao R., Yan Y., Chen S., You H., Xing J., Dong Y. (2019). Transgelin 2 Promotes Paclitaxel Resistance, Migration, and Invasion of Breast Cancer by Directly Interacting with PTEN and Activating PI3K/Akt/GSK-3β Pathway. Mol. Cancer Ther..

[64] Berge G., Mælandsmo G.M. (2010). Evaluation of potential interactions between the metastasis-associated protein S100A4 and the tumor suppressor protein p53. Amino Acids.

[65] Orre L.M., Pernemalm M., Lengqvist J., Lewensohn R., Lehtiö J. (2007). Up-regulation, Modification, and Translocation of S100A6 Induced by Exposure to Ionizing Radiation Revealed by Proteomics Profiling. Mol. Cell. Proteom..

[66] Blagih J., Zani F., Chakravarty P., Hennequart M., Pilley S., Hobor S., Hock A.K., Walton J.B., Morton J.P., Gronroos E. (2020). Cancer-Specific Loss of p53 Leads to a Modulation of Myeloid and T Cell Responses. Cell Rep..

[67] Miller D.M., Thomas S.D., Islam A., Muench D., Sedoris K. (2012). c-Myc and Cancer Metabolism. Clin. Cancer Res..

[68] Seo Y.R., Kelley M.R., Smith M.L. (2002). Selenomethionine regulation of p53 by a ref1-dependent redox mechanism. Proc. Natl. Acad. Sci. USA.

[69] Lin C.-W., Lai G.-M., Chen K.-C., Lin T.-H., Fan J.-J., Hsu R.-L., Chou C.-M., Lin C.-M., Kandaswami C.C., Lee M.-T. (2015). RPS12 increases the invasiveness in cervical cancer activated by c-Myc and inhibited by the dietary flavonoids luteolin and quercetin. J. Funct. Foods.

[70] Chen Z., Xu X. (2016). Roles of nucleolin. Saudi Med. J..

[71] Easton J.B., Houghton P.J. (2006). mTOR and cancer therapy. Oncogene.

[72] New M., Van Acker T., Sakamaki J.-I., Jiang M., Saunders R.E., Long J., Wang V.M.-Y., Behrens A., Cerveira J., Sudhakar P. (2019). MDH1 and MPP7 Regulate Autophagy in Pancreatic Ductal Adenocarcinoma. Cancer Res..

[73] Guha P., Tyagi R., Chowdhury S., Reilly L., Fu C., Xu R., Resnick A.C., Snyder S.H. (2019). IPMK Mediates Activation of ULK Signaling and Transcriptional Regulation of Autophagy Linked to Liver Inflammation and Regeneration. Cell Rep..

[74] Sharma K., Le N., Alotaibi M., Gewirtz D.A. (2014). Cytotoxic Autophagy in Cancer Therapy. Int. J. Mol. Sci..

